# Cardiac Regeneration After Myocardial Infarction: an Approachable Goal

**DOI:** 10.1007/s11886-020-01361-7

**Published:** 2020-08-10

**Authors:** Mauro Giacca

**Affiliations:** 1 King’s College London, British Heart Foundation Centre of Research Excellence, School of Cardiovascular Medicine & Sciences, SE5 9NU London, United Kingdom; 2 Department of Medical, Surgical and Health Sciences, University of Trieste, 34127 Trieste, Italy

**Keywords:** Cardiomyocyte, Myocardial infarction, microRNA, Lipid nanoparticle, Regeneration, Stem cells

## Abstract

**Purpose of Review:**

Until recently, cardiac regeneration after myocardial infarction has remained a holy grail in cardiology. Failure of clinical trials using adult stem cells and scepticism about the actual existence of such cells has reinforced the notion that the heart is an irreversibly post-mitotic organ. Recent evidence has drastically challenged this conclusion.

**Recent Findings:**

Cardiac regeneration can successfully be obtained by at least two strategies. First, new cardiomyocytes can be generated from embryonic stem cells or induced pluripotent stem cells and administered to the heart either as cell suspensions or upon ex vivo generation of contractile myocardial tissue. Alternatively, the endogenous capacity of cardiomyocytes to proliferate can be stimulated by the delivery of individual genes or, more successfully, of selected microRNAs.

**Summary:**

Recent experimental success in large animals by both strategies now fuels the notion that cardiac regeneration is indeed possible. Several technical hurdles, however, still need to be addressed and solved before broad and successful clinical application is achieved.

## Introduction

The need to develop novel therapies for heart failure (HF) consequent to myocardial infarction (MI) is impelling. Despite notable progress in the application of devices assisting the failing myocardium [1], HF prognosis remains poor, with mortality estimated at 40% of patients at only 4 years from diagnosis [2]. This is worse than several common cancers. HF is also tremendously expensive, representing 2–3% of national health expenditures in high-income countries, projected to more than doubling in the next 20 years [3, 4].

Most notably, pharmacological treatment of HF uses drugs that have only marginally evolved since the mid-1990s. While high hope is now raised by the unpredicted and somehow surprising cardiovascular effects of SGLT2 inhibitors [5], for which no convincing molecular explanation yet exists, no conceptually novel drugs have been introduced in the management of patients with HF since the angiotensin II receptor blockers [6]. The relatively novel angiotensin receptor-neprilysin inhibitor (ARNI) combination [7] is based on drugs that were both individually developed in the 1990s. In addition, a number of drugs have so far failed in Phase III clinical trials [8]. Even more remarkably, for conditions that are as prevalent as MI and HF, no biological therapy has yet been developed, based on any protein, peptide, antibody, or nucleic acid [9].

### The Problems of Cardiomyocyte Loss

It has become progressively clear that a major problem underlying the prevalence of HF is linked to the ageing of the population and the lack of regenerative potential of the heart. Acute myocardial injury can kill as many as 25% of cardiomyocytes (CMs) from the left ventricle, corresponding to up to 1 billion cells [10]. In addition, chronic myocardial disease can kill CMs over prolonged periods of time. This is now clear in a number of pathological conditions, ranging from inherited cardiomyopathies to oncological treatments [11]. CM loss also accompanies physiological ageing [12].

This progressive or sudden loss of contractile cells during life is not paralleled by significant new contractile tissue formation. At least three different types of information are concordant in indicating that the extent of CM renewal in adult life is minimal and certainly clinically negligible. First, ^14^C-carbon dating of human CM DNA indicated that renewal of these cells in a 70-year-old individual is less than 50% [13], in fact showing that the majority of CMs at any time in adulthood are those generated at birth or immediately afterwards. Second, measurements obtained using mass spectrometry imaging in mice revealed a rate of CM renewal of approximately 1% per year, which raises three times after MI [14]. These values are consistent with those detected by ^14^C dating. Third, the same information was also obtained by analysing the rate of DNA synthesis in vivo in mice [15].

Lack of CM renewal reflects the incapacity of CMs to replicate. CM replication occurs during embryonic, foetal, and immediate post-natal life, to eventually drop down suddenly in the early neonatal stage [16]. As a consequence, immediately after birth, CM can replicate and drive cardiac regeneration, while this property is irreversibly lost at 7 days in mice. Similar observations also hold true in pigs, in which MI is repaired by complete regeneration in 2-day-old piglets, while invariably leads to scarring in adult animals [17]. An anecdotical report in an infant with acute MI—a very rare condition—reveals that complete cardiac regeneration is also possible immediate after birth in humans [18].

The incapacity of the mammalian heart to regenerate in adulthood contrasts with the evidence in amphibians and fish, in which the regenerative capacity persists throughout life [19, 20]. Of note, regeneration in these animals, similar to neonatal mice and pigs, does not involve the proliferation and differentiation of any cardiac stem cell, but is sustained by the partial de-dedifferentiation of existing CMs that resume proliferation [21, 22]. In adult mice, there is also an attempt at proliferation by CMs bordering the infarcted region [14, 15]; however, this is abortive and the extent of new CM formation is largely below the threshold necessary to provide clinical benefit.

Over the last years, there has been intense research to unravel the reason why CM proliferation stops irreversibly after birth. A prevalent view is that this is linked to sudden biochemical and mechanical events occurring immediately after birth. Pressure overload [23], sudden increase in oxygen tension and oxidative stress [24], lack of maternal factors [25], changes in hormonal stimulation [26], and switch from glycolytic to oxidative metabolism [27] are all factors that have been associated with the rapid loss of regenerative capacity. Most reasonably, it appears likely that the withdrawal of CMs from the cell cycle and the activation of a hypertrophic gene programme is the consequence of a combination of all these factors.

### Factors and Pathways Regulating Cardiomyocyte Proliferation

**FIG. 1:**
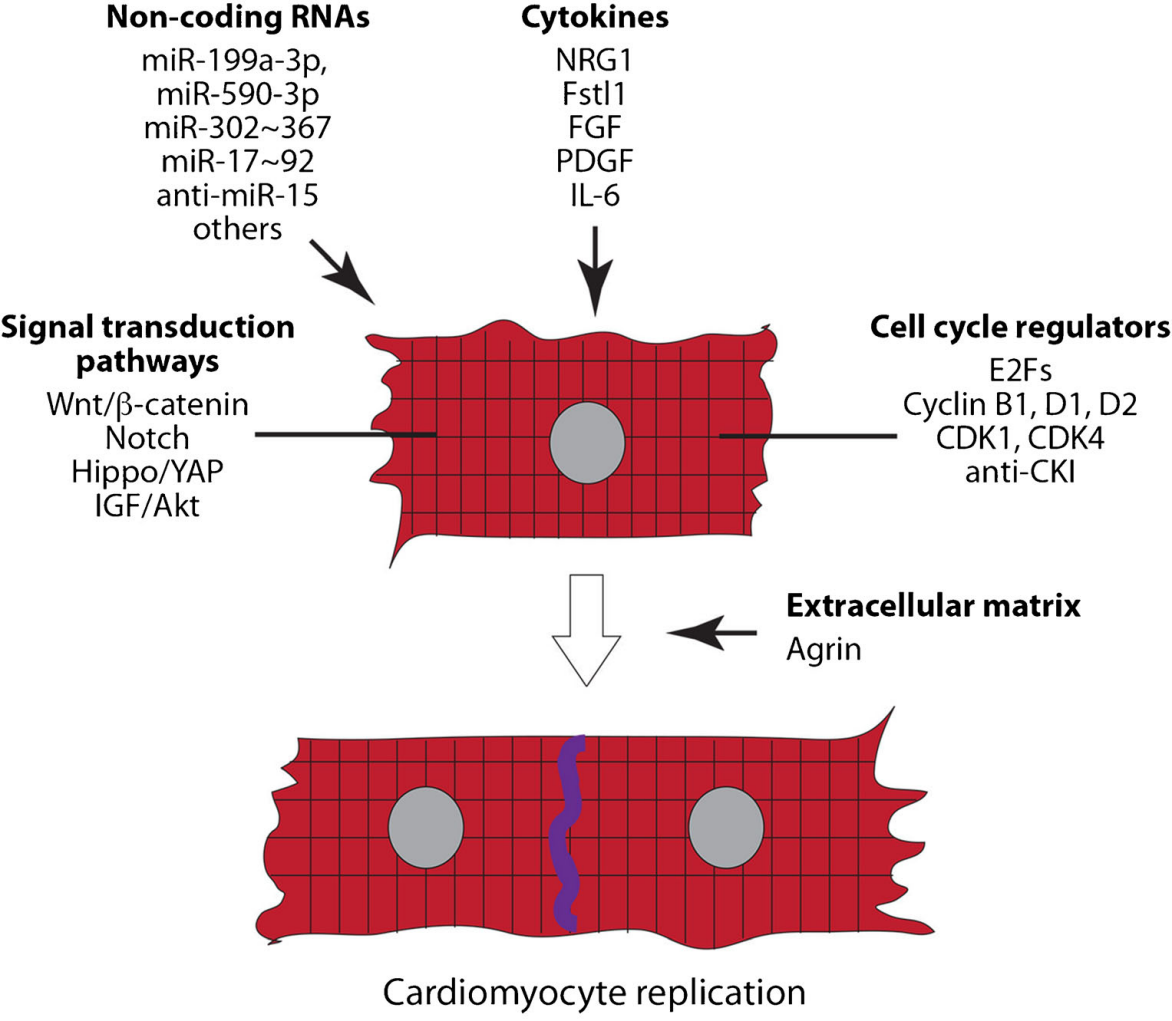
Intracellular and extracellular proteins, genes, and non-coding RNAs known to regulate cardiomyocyte proliferation and cardiac regeneration. See text for explanation

Not unlikely all other cell types, the regulation of CM proliferation occurs through the combined action of a number of factors signalling to CMs from the outside of the plasma membrane or transducing their proliferative signal intracellularly (Fig. 1). These will be briefly reviewed in this section.

#### Growth Factors and Their Receptors

Several growth factors have been identified that can stimulate CM proliferation, especially in conditions when proliferation already occurs spontaneously such as during the embryonic and neonatal stages. These include interleukin-6 (IL-6) [28, 29], platelet-derived growth factor (PDGF) [30], members of the fibroblast growth factor (FGF) family [31, 32], follistatin-like 1 (Fstl1) [33], and neuregulin-1 (NRG1) [34–36]. Paracrine control of CM proliferation was also reported for factors secreted by T-regulatory cells [25] and cardiac resident monocytes [37]. Finally, CM replication was also recently reported to respond to changes in the extracellular matrix, which are mediated agrin [38]. In particular, this factor, which is a component of the neonatal ECM, appears to stimulate CM division through a mechanism that involves the disassembly of the CM membrane-associated dystrophin-glycoprotein complex (DGC) [38].

#### Signal Transduction Pathways

At least three major signal transduction pathways were shown to participate in the regulation of CM proliferation during the embryonic, foetal, and neonatal life. During embryonic development, CM proliferation is regulated by the Wnt/β-catenin pathway. In the absence of Wnt, the levels of cytoplasmic β-catenin are low since this protein is targeted for degradation by a destruction complex also including glycogen synthase kinase 3β (GSK-3β) [39]. In CMs, inhibition of GSK3-β, through genetic means [40] or by treatment with the inhibitory small molecule BIO [41], results in β-catenin stabilization and translocation to the nucleus, where the protein acts as a transcriptional coactivator of members of the T cell factor (TCF)/Lymphoid enhancer factor (LEF) family of proteins, followed by CM proliferation.

A second signal transduction mechanism relevant to CM proliferation is the Notch pathway. Notch signalling requires cell–cell contact and occurs through engagement of the Notch receptor to one of its ligands, in particular, Jagged1 in the mammalian heart, followed by translocation of the cleaved Notch intracellular domain (ICD) to the nucleus, where it acts as a transcriptional coactivator [42]. Notch regulates proliferation of immature CMs during the foetal and post-natal life [43–45].

A third essential pathway involved in CM proliferation converges in the activation of another transcriptional cofactor, the YAP protein, and its cognate factor TAZ. This is the final positive effector of the inhibitory Hippo pathway. In resting CMs, YAP is maintained inactive by phosphorylation of a kinase cascade, which prevent its nuclear translocation and drives its proteolytic degradation. These kinases include LATS1/2 with its cofactor MOB1 and MST1/2 (Hippo in Drosophila) with its cofactor SAV1. Other inhibitory kinases are TAOK1 and STKL38. Regulation of the Hippo pathway is a main mechanism for CM mechanosensing, which transduces stretch and tension signals from the extracellular environment into the cell nucleus. Genetic deletion of MST1, SAV1, and LATS results in cardiac hyperplasia [46], while overexpression of MST1 [47] or LATS2 [48] leads to post-natal dilated cardiomyopathy. Transgenic overexpression of a constitutively active YAP mutant (YAPS112A) causes CM hyperproliferation [49, 50].

#### Cell Cycle Regulators

Similar to all cell types, cell cycle regulation in CMs is also governed by a series of positive and negative regulators that converge on cyclin/CDK activation. Work performed over the last several years has shown that overexpression of E2F transcription factor [51–53] or cyclin D1 and D2 [54–56] can lead to CM proliferation. Similar results can be achieved by knocking down the cyclin-dependent kinase inhibitors p21^WAF1/CIP1^, p27^KIP1^, and p57^KIP2^ [57] or Meis1, a homeodomain transcription factor that activates expression of p16^INK4a^ and p21^WAF1/CIP1^ [58]. The observation that transgenic animals overexpressing cyclin A2 [59, 60], cdk2 [61], cyclin D1 [54], and cyclin D2 [62, 63] have increased CM proliferation is also consistent with these findings.

### Stem Cells and Genes for Cardiac Regeneration

Starting from the early 2000s there has been a massive interest in developing innovative strategies for cardiac regeneration. Fuelled by the apparent existence of stem cells in multiple tissues from adult individuals, several clinical attempts were based on the injection, into the infarcted or failing heart, of cells recovered from the bone marrow (unfractionated, immunopurified for c-kit antigen expression or expressing markers of stromal mesenchymal cells [64, 65]) or purified from the heart itself [66, 67]. Despite marginal evidence of clinical benefit in some of these studies due to paracrine effects [68, 69], the current overall consensus is that no proof of actual cardiac regeneration (i.e. formation of new cardiomyocytes and cardiac tissue) was achieved by any of these studies. More in general, no convincing evidence exists that stem cells of any derivation from adult individuals exist that might regenerate the heart [70]. Examination of some of the original studies with c-kit-positive cells has led to the retraction of some of the published findings (http://circ.ahajournals.org/content/129/16/e466.full.pdf+html) and investigation on the integrity of others (10.1016/S0140-6736(14)60608-5).

Embryonic stem cells or iPS cells can be expanded in the laboratory to generate a number of CMs, in the order of 0.5–1 billion, to be directly injected into the heart. This approach has successfully translated into improved cardiac repair in a series of recent studies in infarcted monkeys infused with these cells into the infarcted area [71, 72••]. A more complex approach along the same theme is the ex vivo generation of large patches of myocardial tissue starting form hiPS- or hES-derived CMs based on their integration into 3D contractile tissue [73•, 74]. This is a promising approach to generate contractile tissue, thanks to the spontaneous property of CMs to organize into myocardial-like tissue once subjected to load in the laboratory. The concept of substituting lost cardiac tissue by infusing individual CMs or ex vivo generated cardiac tissue is of intuitive interest, however not devoid of a series of problems. The cells that can be obtained to date from hiPS or hES cells have an embryonic phenotype and generate arrhythmias once injected into the heart; the recipients need to be immunosuppressed to avoid rejection and integration into the cardiac electric and mechanical syncytium is still imperfect. More notably, both CM infusion and cardiac tissue implantation are complex procedures in their nature, which contrasts with the broad demand for cardiac regeneration.

A possibly simpler and translatable possibility is to re-awaken the endogenous potential of CMs to proliferate and thus mimic what spontaneously occurs in the neonatal mammalian heart or in amphibians and fish throughout life. This is quite efficient in transgenic mouse models. For example, mice transgenic for activated YAP or knock out for the inhibitor Mst1 kinase or for the Mst1 co-factor Salvador can regenerate the heart after MI [50, 75, 76••]. For some of the activators, regeneration was also reported when their genes were administered using viral vectors. Among others, this was reported for YAP [77] or the Notch intracellular domain [43, 45]. However, exogenous delivery is not fraught with problems. Permanent overexpression YAP needs to be taken with caution, given the tumour suppressor role that the Hippo pathway exerts in several cancers [78]. In the case of Notch, this pathway is instead inactivated in adult hearts by suppressive epigenetic modifications at Notch-responsive promoters [79]. Finally, CM replication and cardiac regeneration was shown to be achievable by the simultaneous delivery of multiple cell cycle regulators [80]; this approach, however, appears quite problematic for therapeutic applications.

### Stimulation of Endogenous Cardiac Regeneration by microRNAs

An appealing strategy to achieve cardiac regeneration is to manipulate the CM proliferative potential by microRNAs (miRNAs). These short, double-stranded RNA molecules control virtually any aspect of cell biology, including proliferation. This also holds true for CMs (reviewed in ref. [81]). The miRNAs stimulating endogenous CM proliferation can be classified into one of three categories. First, several pro-regenerative miRNAs include molecules that are highly expressed in ES cells and are required to maintain pluripotency. These include members of the miR-302~367 and miR-miR-290 families, which share the same seed sequence [82, 83]. Activation of the miR-302-367 cluster after MI in mice induces cardiac regeneration, as does the transient delivery of some of its members as synthetic molecules [84]. A second group of miRNAs that can induce CM regeneration includes a series of miRNAs involved in tumorigenesis. These include the miR-17~92 cluster (also named OncomiR1) [85, 86], and its paralogue clusters miR-106b~25 and miR-106a~363 [87, 88]. Also, in this case, both transgenic expression of the miR-17~92 cluster cluster [89] or the administration of the cluster member miR-19a/19b [90] led to cardiac regeneration. A third group of miRNAs was identified through two large screenings of human miRNAs [91, 92]. The most prominent and studied of these miRNAs is miR-199a-3p, which was shown to stimulate cardiac repair both when expressed from an AAV9 vector [91] or a synthetic RNA molecule [93] in mice and from an AAV6 vector in pigs [94••]. Several other miRNAs are also capable, to a various extent, to induce cardiomyocyte proliferation (extensively reviewed in ref. [81]), indicating that the withdrawal of CMs from the cell cycle at birth is not an irreversible process but a regulated one, with the possibility of reverting it through the manipulation of gene expression.

The identification of miRNAs with the potential to stimulate cardiac regeneration appears particularly appealing for translational purposes, as these molecules can be developed as treatments fitting virtually all patients and do not require extensive ex vivo manipulation, as instead is required for stem cell applications. MicroRNAs can be delivered either as expressed from viral vectors or as synthetic molecules. In the former case, AAV vectors now appear as the vectors of choice for cardiac gene delivery [95]. Using AAVs to deliver genes promoting cardiac regeneration, however, is not devoid of problems. These vectors persist virtually indefinitely in CMs, while, to date, no promoter has been described in humans or non-human primates from which expression can be turned off. This creates obvious safety issues and efficacy concerns as far as CMs are concerned, as the stimulation of proliferation in these cells requires the transient disassembly of the sarcomeric apparatus and cell dedifferentiation [22, 96, 97]. There appears to be a need, therefore, to apply the pro-proliferative stimulus transitorily. In addition, endogenous transcription of a miRNA gene is followed by processing of the pri-miRNA by the cellular RNAi machinery. This leads to the generation of both miRNA strands, which can be not necessarily desirable. These problems surfaced in the only large animal study so far performed to test the efficacy of one miRNA, miR-199a, to drive cardiac regeneration in pigs [94••]. In this study, cardiac regeneration and improvement of cardiac function was very significant at 1 month after treatment of infarcted pigs; however, several animals developed fatal arrhythmias at longer times. The hearts of the treated animals showed the presence of proliferating and undifferentiated cells, along with the expression of both the pro-regenerative miR-199a-3p strand but also of its complementary miR-199a-5p strand, which is known to exert undesirable effects in the heart [94••].

One of the possible strategies to overcome this limitation of AAV-mediated gene delivery is to resort to the possibility of administering the miRNAs as synthetic RNA molecules. Already available evidence shows that a single intramyocardial injection miR-199a-3p or miR-590-3p mimics using a lipofectamine-based formulation can stimulate a regenerative response in mice [93]. Similar results were also obtained by a single intramyocardial injection of miR-19a/19b [90] or miR-302b/c mimics [98] or the daily intravenous administration of miR302b/c [84], miR-19a/19b [90], or miR-708 [99].

## Conclusions

The notion that regeneration of the cardiac muscle can be achieved by either the implantation of ex vivo generated CMs or through the stimulation of the proliferative capacity of endogenous cells to proliferate remains exciting. It challenges a long-standing dogma that damage in post-mitotic tissue is intrinsically irreversible and offers hope for treatment to the vast number of patients with post-ischemic HF. Both the stem-cell and the endogenous regeneration approaches, however, still require significant improvement before extensive clinical application. In the case of stem cells, ES- or iPS-derived CMs are still immature, their electrical and mechanical coupling to endogenous CM is limited and the process of deriving the large number of cells required for each patient is demanding for scaling up. Maturation appears improved when CMs are used to form ex vivo, 3D cardiac tissue; however, the problem of integrating large patches of engineered myocardium into the preexisting one still remains.

In the case of endogenous cardiac regeneration, this approach appears more amenable to application in a large number of patients and less demanding in terms of development. However, a main problem remains the efficacy of delivery of genes or miRNAs to the infarcted myocardium. The experiments with lipid-mediated delivery in mice appear promising, but translation of these findings into large animals will require extensive experimentation. In this respect, it is however exciting to note that the field of small RNA delivery has made substantial progress over the last decade, with the generation of lipid nanoparticles (LNPs) with neutral surface charge, and of methods to load small nucleic acids into these particles [100]. The first LNP delivering an RNAi therapeutic molecule to reach the market was patisiran in 2018—this drug lowers the hepatic levels of transthyretin for the treatment of hereditary transthyretin-induced amyloidosis [101]. Several other LNP formulations of siRNAs and miRNAs for other applications are in the pipeline of clinical experimentation. Whether an LNP delivering one of the pro-proliferative miRNAs for CMs might be effective in inducing cardiac regeneration in large animals and is thus amenable to clinical translation awaits the results of ongoing experimentation.
